# Insights into the Cardiotoxic Effects of *Veratrum Lobelianum* Alkaloids: Pilot Study

**DOI:** 10.3390/toxins14070490

**Published:** 2022-07-15

**Authors:** Amir Taldaev, Roman P. Terekhov, Elizaveta V. Melnik, Maria V. Belova, Sergey V. Kozin, Andrey A. Nedorubov, Tatyana Ya. Pomerantseva, Galina V. Ramenskaya

**Affiliations:** 1 Laboratory of Nanobiotechnology, Institute of Biomedical Chemistry, 119121 Moscow, Russia; 2 A.P. Nelyubin Institute of Pharmacy, I.M. Sechenov First Moscow State Medical University (Sechenov University), 119991 Moscow, Russia; terekhov_r_p@staff.sechenov.ru (R.P.T.); melnik_e_v_2@staff.sechenov.ru (E.V.M.); belova_m_v@staff.sechenov.ru (M.V.B.); kozin_s_v@staff.sechenov.ru (S.V.K.); nedorubov.ras@gmail.com (A.A.N.); pomerantseva_t_ya@staff.sechenov.ru (T.Y.P.); ramenskaya_g_v@staff.sechenov.ru (G.V.R.); 3 N.V. Sklifosovsky Research Institute for Emergency Medicine, 129090 Moscow, Russia

**Keywords:** *Veratrum*, alkaloid, jervine, protoveratrine, cardiac sodium channel, molecular docking, machine learning-based SAR, case series, intoxication, HPLC-MS/MS

## Abstract

Jervine, protoveratrine A (proA), and protoveratrine B (proB) are *Veratrum* alkaloids that are presented in some remedies obtained from *Veratrum lobelianum*, such as *Veratrum aqua*. This paper reports on a single-center pilot cardiotoxic mechanism study of jervine, proA, and proB in case series. The molecular aspects were studied via molecular dynamic simulation, molecular docking with cardiac sodium channel Na_V_1.5, and machine learning-based structure–activity relationship modeling. HPLC-MS/MS method in combination with clinical events were used to analyze *Veratrum* alkaloid cardiotoxicity in patients. Jervine demonstrates the highest docking score (−10.8 kcal/mol), logP value (4.188), and p*K_a_* value (9.64) compared with proA and proB. Also, this compound is characterized by the lowest calculated IC_50_. In general, all three analyzed alkaloids show the affinity to Na_V_1.5 that highly likely results in cardiotoxic action. The clinical data of seven cases of intoxication by *Veratrum aqua* confirms the results of molecular modeling. Patients exhibited nausea, muscle weakness, bradycardia, and arterial hypotension. The association between alkaloid concentrations in blood and urine and severity of patient condition is described. These experiments, while primary, confirmed that jervine, proA, and proB contribute to cardiotoxicity by Na_V_1.5 inhibition.

## 1. Introduction

Alkaloids are a group of biologically active basic compounds widely occurring in animal and plant sources [1,2,3]. They find great application in ethnomedicine and traditional Chinese medicine [4,5]. Moreover, alkaloids demonstrate potential in modern research as promising objects for drug design [6,7,8].

More than 200 alkaloid molecules are found in *Veratrum* spp. [9]. Based on their chemical structures, these compounds are divided into two groups [10]. Alkaloids containing the typical cyclopentanoperhydrophenanthrene core are called *Solanum* alkaloids. Another group, named *Veratrum* alkaloids, is characterized by nor-homo-transformation of C and D rings.

*Veratrum lobelianum* belongs to the family Melanthiaceae. It is a Eurasian species, occurring from Central Europe to the Russian Far East [11]. This plant is also poisonous. Nevertheless, in Russia it is used as a folk remedy against alcoholism [12], that often results in intoxication cases. More than that, *V. lobelianum* is monographed in Russian pharmacopeia and used for *Veratrum aqua* production. The last one is an alcoholic tincture of rhizomes and roots, diluted by water twice that is labeled for topical application in pediculosis treatment. The same pharmacological activity is known for other *Veratrum* spp. [13,14]. This drug is used off-label *per os* by alcohol-addicted persons.

The major alkaloid components of *Veratrum aqua* are jervine, protoveratrine A (proA), and protoveratrine B (proB), that are classified as *Veratrum* alkaloids [15]. Nevertheless, the presence of these alkaloids is not taken into account in *Veratrum aqua* specification. However, the *Veratri lobeliani rhizomata cum radicibus* are standardized by the sum of alkaloids calculated with reference to protoveratrine, using acid-base titration [16]. According to the literature data, jervine demonstrates antitumor [17,18], anti-inflammatory and analgesic [19], as well as radioprotective [20] activity. The pharmacology of protoveratrines and their derivatives was out of scope in last 10 years. However, it is known that the sum of alkaloids affects heart chemoceptors and motoceptors, blocking conductivity. The increase in sodium channel permeability was also observed, resulting in depolarization of neurons and cardiocytes. What is not yet clear is the contribution of each separate *Veratrum* alkaloid on cardiotoxicity. To our knowledge, the molecular mechanism of this process has not been identified.

This paper reports on a pilot cardiotoxic mechanism study of jervine, proA, and proB, which was performed by molecular modeling with cardiac sodium channel Na_V_1.5 and observation in emergency patients poisoned by *Veratrum aqua*.

## 2. Results

### 2.1. Molecular Modeling

The first set of experiments includes computational calculations of several physicochemical and biochemical parameters of *Veratrum* alkaloids, applying molecular dynamic (MD) simulation, molecular docking, and machine learning-based structure–activity relationship (SAR) modeling.

**Figure 1:**
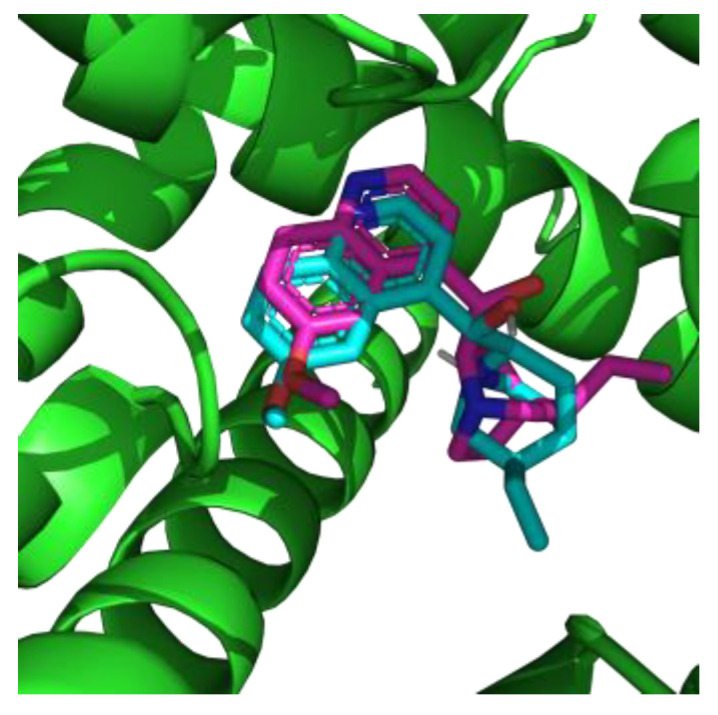
Superposition of native (cyan) and re-docked (magenta) conformations of quinidine into the Na_V_1.5 binding site.

Cardiac sodium channel Na_V_1.5 was used as a biological target and quinidine served as a reference compound with approved affinity to this integral membrane protein. Conformation of re-docked quinidine was similar to experimental structure (Figure 1). Root-mean-square deviation between re-docked and experimental poses was 2 Å. This allowed us to validate the applicability of the chosen docking method.

Table 1 provides the summary of in silico analysis. Root-mean-square errors for the training and the test sets in machine learning-based SAR were 0.072 and 0.329, correspondingly. Coefficients of determination (*R*^2^) were also calculated. It was 0.982 for the training set and 0.629 for the test set. These parameters of machine learning-based SAR demonstrate their applicability for the research purposes. What stands out in the table is the comparable predicted IC_50_ value for quinidine and *Veratrum* alkaloids. Jervine demonstrates the highest docking score, logP value and p*K_a_* value, that, probably, results in the lowest predicted IC_50_ value. At the same time, quinidine is characterized by intermediate data values.

**Figure 2:**
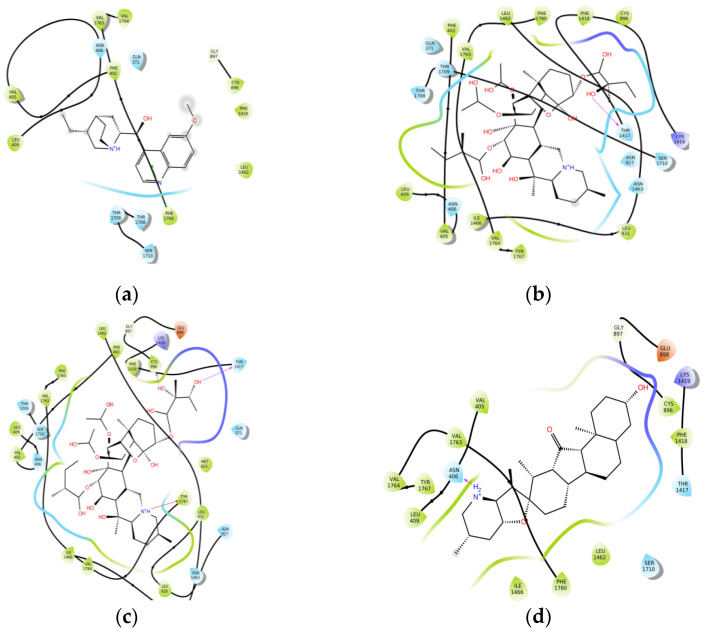
Interaction maps between investigated compounds and amino acid residues of sodium channel: (**a**)—quinidine; (**b**)—proA; (**c**)—proB; (**d**)—jervine.

Generally, *Veratrum* alkaloids and quinidine interact with Na_V_1.5, mainly van der Waals forces, due to their steroidal structure (Figure 2). We did not find any hydrogen bond occurrence with sodium channel. *Veratrum* alkaloids form 1–3 hydrogen bonds with amino acid residues of protein. The low affinity of proA could be explained by the unfavorable hydrogen bond of the charged amine group with Asn 406 amino acid residue of the side chain.

The results of in silico study demonstrate that, apparently, jervine, proA, and proB have molecular mechanism of action to do with quinidine. These data were in need of confirmation by an in vivo experiment.

### 2.2. Clinical Study

Turning now to clinical evidence on the cardiotoxic mechanism of *Veratrum* alkaloids, the following data are presented. 

Seven cases of *Veratrum aqua* intoxication were involved in this study. There were 4 Caucasian males and 3 Caucasian females. Patients’ ages varied from 20 to 74 years, the median age was 46. Case histories included accidental or suicidal 50–100 mL intake of *Veratrum aqua per os*. 

In 2 cases, the intake time was unknown. One patient had taken *Veratrum aqua* 4–5 h before admission to hospital. Finally, in 4 cases patients were hospitalized 2–3 h after tincture administration. The primary signs of intoxication were hypersalivation, nausea, multiple vomiting with abdominal pain, muscle weakness, and bradycardia, that were observed at the pre-admission phase. To reduce these symptoms the intravenous atropine solution (0.1%), prednisolone, and fluid therapy were used. 

All patients were hospitalized in critical but stable condition. Clouded consciousness and coordination dysfunction were observed after admission. The other symptoms included muscle weakness, numbness in the limbs, pearlescence, decreased heat rate (42–50 bpm), and arterial hypotension (60/40–100/60 mm Hg). In one case (patient 3) bradycardia (50 bpm) was registered in addition to short-term hypertension (140–165 mm Hg) with a subsequent decrease of arterial pressure that lasted over 4 h. Interestingly, through the consequent chemico-toxicological analysis this case was characterized by highest concentration of *Veratrum* alkaloids in blood plasma.

The use of qualitative and quantitative HPLC-MS/MS analysis of *Veratrum* alkaloids in biofluids gave an opportunity to confirm intoxication cause (Table 2). This method was validated for qualitative and quantitative analysis of jervine and proA; also, proB could be detected this method, while the selectivity for this compound was approved. Furthermore, in combination with clinical events, this method served to control the severity of a patient’s condition. According to Rokin and Sentsov classification [22], based on the clinical events of intoxication by remedies on *Veratrum* basis, the severity of the condition of the majority of observed patients can be characterized as moderate, except case 3, which demonstrated major severity. In 4 cases, the intoxication symptoms disappeared in 24 h, while alongside the concentrations of *Veratrum* alkaloids decreased to the limit of detection (0.1 ng/mL). Jervine concentration in another 2 patients decreased by 71–73% at the end of 24 h, and it was fully eliminated from blood in 36 h. ProA and proB had been observed on admission and were not detected at the end of the first day. What is more, in case 3 the decrease of jervine concentration to the detection limit occurred in 70 h, proB was detected during 36 h, and proA concentration was determined at 24 h only. The correlation between the decrease in *Veratrum* alkaloid concentration and regression of clinical condition severity was quite obvious in this case. All patients were characterized by asthenia up to day 3. The mean time of stay at the emergency department was 48 h. Hospitalization took from 3 to 6 days. Patient 3 was kept at the emergency department for 78 h. 

Patient management included detoxification infusion therapy and forced diuresis under water-electrolyte balance control. To reset the heart rate, the intravenous atropine solution (0.1%) was additionally used. Dexamethasone, tocopherol, unithiol, cordiamine, and potassium chloride in 5% glucose intravenous solution comprised cardioprotective therapy. Vitamins (B and C) and symptomatic therapy were also used. 

To assess the efficacy of detoxification, the monitoring of *Veratrum* alkaloids concentration in urine was performed by HPLC-MS/MS analysis (Table 3). The maximum concentrations of proA were observed at the beginning of therapy; however, this parameter varies highly in patients. At the end of 24 h, the proA concentration considerably reduced up to 35 times. ProB was observed in urine for a longer time compared with a previous alkaloid. Excretion profile of jervine remarkably differs compared with proA and proB. Firstly, the concentration of this alkaloid varies from 0.21 to 1.71 ng/mL. At the end of 24 h, the concentration of jervine in patients’ urine increased notably up to maximum and then decreased. 

In general, the severity of patients’ conditions correlated with the concentrations of jervine in blood plasma. This is a rather remarkable outcome of the clinical study. The monitoring of *Veratrum* alkaloids in urine demonstrates the efficacy of detoxification.

## 3. Discussion

The present study was designed to determine the molecular mechanism of *Veratrum* alkaloid cardiotoxic effect.

Molecular modeling gives an opportunity to predict the biological activity level and bioavailability of small molecules [23,24,25]. Quinidine was chosen as a reference compound due to its antiarrhythmic effect resulting in Na_V_1.5 inhibition [26]. The correlation between docking score and predicted IC_50_ value was observed. Coherence of data obtained by different methods confirms its reliability. Jervine is characterized by the highest IC_50_ value, suggesting pronounced biological effects.

The structures of supramolecular complexes of jervine, proA, proB, and quinidine with Na_V_1.5 were predicted by molecular docking. Apparently, all analyzed compounds bind with biological targets by van der Waals forces. At the same time, polar intramolecular interactions are also observed, that increase the binding efficacy. The similar behavior is known for local anesthetics [27,28]. Procaine, lidocaine, articaine etc. contain hydrophobic core and hydrophilic amino group, both being pharmacophores.

**Figure 3:**
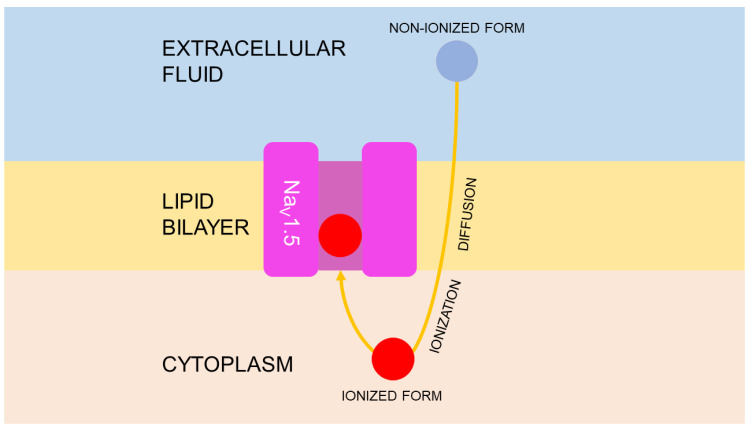
Summary of action mechanism of *Veratrum* alkaloids. It is similar to local anesthetics and 1A class of antiarrhythmic drugs.

Transmembrane permeability is another important characteristic for realization of pharmacological effects. Simple diffusion is the only way of membrane transport for local anesthetics. The rate of this process correlates with p*K_a_* value. Hence, lidocaine (p*K_a_* value is 7.9) acts fast, while bupivacaine (p*K_a_* value is 8.1) demonstrates suspended action onset [29,30]. Based on p*K_a_* values calculated by computational methods, it is possible to suggest that *Veratrum* alkaloids are ionized in the cytoplasm and bind with Na_V_1.5 in ionized form. It is known that the pH value of extracellular fluid is 7.4. The increased p*K_a_* values decrease a share of non-ionized molecules, which have the ability to cross membrane. Based on this parameter prediction, jervine, possibly, has a lower constant of transmembrane diffusion than proA and proB. It appears that the percent of non-ionized form of this compound in tissues is 0.6% only. Therefore, not more than 0.6% of jervine molecules may cross the membrane of nerve cells to bind with Na_V_1.5 in ionized form. However, jervine is characterized by a higher logP value that could promote transmembrane permeability. Additionally, affinity of this alkaloid was quite high, that makes sense for Na_V_1.5 inhibition. At the same time, the percent of non-ionized forms of proA and proB was 65.3%. Due to high concentration in cytoplasm, these compounds may have a great impact on Na_V_1.5 inhibition, despite lower affinity to this biological target, compared with jervine. As a result, the cardiotoxic effect of *Veratrum aqua* most likely is associated with all three studied alkaloids of *V. lobelianum*. Summarizing these data, the mechanism of cardiotoxic action of *Veratrum* alkaloids was suggested (Figure 3).

The seven patients of this study suffered from off-label intake of *Veratrum aqua* with multiple cardiovascular and neurological disorders and general intoxication. This observation is in line with other reports [31,32,33]. The wide range of patients’ age and presence of both male and female patients in the observed group are the advantages of this study. Notably, our clinical findings are quite similar with quinidine side effects resulting through overdosing, which is common due to a narrow therapeutic window [34,35]. It includes decreased heat rate, arterial pressure decrease, and atrioventricular block [36]. The similarity of clinical patterns provides further support for the identity of the cardiotoxic mechanism of *Veratrum* alkaloids and the reference compound.

In our research, the presence of jervine, proA, and proB was confirmed by validated analysis by HPLC-MS/MS, developed previously [37]. This instrumental method is characterized by high selectivity and sensitivity and through these advantages finds a great application in chemical analysis of complex objects [38,39,40]. The correlation between *Veratrum* alkaloid concentration and intoxication clinical findings was observed, that confirms the results of in silico study. Thus, the qualitative and quantitative HPLC-MS/MS analysis was translated into “real-life” clinical practice. According to excretion profiles of analyzed alkaloids, it may be concluded that definite diagnosis of *Veratrum aqua* intoxication on the HPLC-MS/MS basis may be performed 24 h after drug intake. The implementation of this HPLC-MS/MS method will provide more information for physicians to support clinical decision on patient management.

The unusual excretion profile of jervine after oral administration was reported previously by Zheng et al. in rats [17]. The presence of two peak concentrations was explained by fast absorption of this alkaloid in gastrointestinal tract that was associated with the first maximum (2 h after intake) and by enterohepatic circulation that caused the second increase of its concentration (in 24 h). The data of molecular docking for jervine with P-glycoprotein and sulfotransferase SULT2A1 supported these findings [41,42]. Apparently, due to the study design the presence of two peak concentrations of jervine in blood was not observed. However, the HPLC-MS/MS analysis of urine demonstrates the similar tendency that can be explained by slow elimination of this alkaloid and its enterohepatic circulation. Furthermore, the longer presence of jervine in blood compared with proA and proB may be attributed to this phenomenon.

This study has some limitations. Results of molecular modeling are appropriate for hypothesis formulation, but they should be supported by in vitro and in vivo findings. The evidence level of case series is not high due to absence of randomization, blinding, and control group [43,44]. Additionally, the observation group in our research was small, racial, and regional representation was not achieved. However, this study design is appropriate for causality evaluation and therefore may be considered suitable a pilot research.

Nevertheless, further work is required to establish the mechanism of biological effects of *Veratrum* alkaloids. Investigations in this area may show the way for the development of new anesthetic and cardiotropic remedies on a medical plant basis.

## 4. Conclusions

The purpose of the current pilot study was to determine the molecular mechanism of cardiotoxic effects of jervine, proA, and proB occurring in *Veratrum aqua*. In order to achieve this goal, the data of molecular modeling and case series were summarized. These experiments, while primary, confirmed that all three alkaloids of *V. lobelianum* contribute to cardiotoxicity Na_V_1.5 inhibition. Jervine demonstrated the highest affinity to biological target, while proA and proB were characterized by better bioavailability. This would be a fruitful area for further work that may result in development of a new herbal treatment while taking into consideration the ways of addressing toxic side effects.

## 5. Materials and Methods

### 5.1. Materials

The following substances were used as a reference sample: jervine (100%, Sigma-Aldrich, Burlington, MA, USA), proA (99%, PhytoLab GmbH & Co. KG, Vestenbergsgreuth, Germany), anhydrous quinidine (88%, Sigma-Aldrich, Burlington, MA, USA). The last one was used as an internal standard. 

Ammonium formate (HPLC-grade) was purchased from Sigma-Aldrich (Burlington, MA, USA) and ammonium hydroxide (chemically pure) was obtained from Chimmed Group (Moscow, Russia). Moreover, methyl *tert*-butyl ether (HPLC-grade, Sigma-Aldrich, Burlington, MA, USA), formic acid (LC-MS-grade, Sigma-Aldrich, Burlington, MA, USA), dimethyl sulfoxide (chemically pure, Chimmed Group, Moscow, Russia), and acetonitrile (HPLC-grade, Avantor Inc., Radnor, PA, USA) were used as solvents.

### 5.2. LogP and pK_a_ Values Calculations for Transmembrane Permeability Prediction

MarvinSketch 21.13 (academic license; ChemAxon Ltd., Budapest, Hungary) with Protonation and Partitioning plugins was used for logP (consensus method) and p*K_a_* (micro mode) values prediction. Molecular models of *Veratrum* alkaloids were uploaded from the ZINC database [45].

### 5.3. Binding Mode and Binding Affinity Predictions by Molecular Docking

The protein structure of human voltage-gated sodium channel Na_V_1.5 was obtained from RCSB Protein Data Bank (PDB ID: 6LQA) [26]. Missing amino acids residues were reconstructed in the SWISS-MODEL web server [46]. We performed a short MD simulation for protein structure relaxation, including side chains. MD was performed in GROMACS 2020.4 software [47]. CHARMM27 force field and TIP3P explicit water model were applied. Macromolecule was centered in a cubic box of periodic boundary conditions of sufficient size, that the minimum distance to period images was 1.0 nm. Sodium and chloride counter ions were added to neutral system net charge in 0.15 M concentration of these ions. The length of MD was 120 ns in NPT microcanonical ensemble (T = 311 K; P = 1 bar) with prior minimization and equilibration MD. Averaged conformation of Na_V_1.5 was extracted from the last 20 ns of MD trajectory with a non-changed root-mean-square deviation of protein-backbone using the gmx cluster module. Small molecules ready-to-docking were fetched from the ZINC database [45]. Protein structure from the MD simulation and ligand molecules were prepared in the AutoDockTools 1.5.6 [48]. Validation of the system by re-docking and molecular docking *Veratrum* alkaloids was performed in the AutoDock Vina 1.1.2 [49]. The Maestro 2021-1 (academic license; Schrödinger, LLC., New York, NY, USA) and the PyMol 2.4.0 (Schrödinger, LLC., New York, NY, USA) molecular graphics systems were used for molecule structures manipulations and image preparations.

### 5.4. Machine Learning-based SAR Modeling

Sample of 266 SCN5A-active compounds was obtained from the ExCAPE chemogenomics database [50]. Cardiac sodium channel Na_V_1.5 is under control of SCN5A gene [51]. The test set size was determined as 10% (27 compounds). Virtual structures of small molecules in SMILES format were represented in RDkit Release_2021.03.4 using 4096-bit Morgan fingerprints [52]. Vector modification of molecules for machine learning and data normalization were performed in NumPy 1.20.3 and scikit-learn v0.23.2, respectively. We chose a feed-forward neural network for our research. Batch size was 30. The network was coded in PyTorch 1.9.1 and contained 3 hidden layers. Each layer consisted of 1024 neurons. Activation function of the neural network ReLU was applied. Epoch number was limited by 500 to avoid the overtraining. Root-mean-square error was used as a loss function. Adam algorithm was selected as stochastic optimization method. Converting of pXC50 values to predicted IC_50_ values conducted accordingly the Equation (1):

IC_50_ = 10^−pXC50^ (1)

### 5.5. Patients

The design of this clinical study is a single-center case series.

From October 2020 to February 2021, we recruited patients from N.V. Sklifosovsky Research Institute for Emergency Medicine in Moscow, Russia. Patients were eligible if they had been admitted to the emergency department with admission notes that included intake of *Veratrum aqua per os* and confirmed presence of *Veratrum* alkaloids in blood. Patients were excluded from the study if they were younger than 18 years.

All patients were diagnosed with *Veratrum* alkaloid intoxication and received the treatment as clinically indicated.

Biofluid samples were collected upon arrival at the emergency department, 24 h after admission and then every 12 h up to 60 h.

### 5.6. Ethics Statement

Human serum and urine samples were used after the patient had signed the informed consent form. The use of human specimens was performed after the protocol was reviewed and approved by the Ethics Committee of N.V. Sklifosovsky Research Institute for Emergency Medicine.

### 5.7. Standard Sample Preparation

Stock solutions (1 mg/mL) of jervine and quinidine standards were prepared by dissolution of 1 mg substance (accurate weight) in 1.00 mL and 0.88 mL of methanol, respectively. Stock solution of proA (1 mg/mL) was obtained using 0.99 mL of dimethyl sulfoxide. 

Reference samples of quinidine were diluted by methanol to get the solutions with following concentrations: 200 µg/mL and 500 ng/mL. The concentrations of the calibration samples of jervine and proA were 0.1 ng/mL, 1.0 ng/mL, 5 ng/mL, 10 ng/mL, 20 ng/mL, 30 ng/mL, 40 ng/mL, 50 ng/mL, and 100 ng/mL.

### 5.8. Sample Preparation

Sample preparation included liquid-liquid extraction using 500 µL of blood plasma or urine and 1 mL of methyl *tert*-butyl ether. The biofluid was alkalinized up to pH value 10 by ammonium hydroxide. Also 25 µL of quinidine (500 ng/mL) were added. This mixture was shaken for 10 min and then centrifuged for 10 min at 3500 rpm. The organic layer was collected and concentrated at room temperature under nitrogen. Dry residue was resolved in 200 µL of acetonitrile. 

### 5.9. HPLC-MS/MS Analysis

Liquid chromatographer Agilent 1260 Infinity II coupled with mass spectrometer Agilent 6460 (Agilent Technologies, Santa Clara, CA, USA) was used for HPLC-MS/MS analysis. The separation was performed using the chromatographic column Poroshell 120 EC-C18 (4.6 mm × 100 mm × 2.7 µm, Agilent Technologies, Santa Clara, CA, USA).

Solvent A and solvent B were used as mobile phase. Solvent A contained 5 mmol/L of ammonium formate in 0.1% water solution of formic acid. Solvent B consisted of 0.1% formic acid in acetonitrile. Gradient elution was performed under the conditions presented in Table 4.

Temperature was set at 45 °C and flow rate in chromatographer was 0.8 mL/min. 

Tandem mass-spectrometric detection was performed in multiple reactions monitoring (MRM) mode. All parameters of analysis were optimized for selected analytes. The following MRM transitions were used:

- Jervine: *m/z* 426.2 > 114.1/109.1/84.1;
- ProA: *m/z* 794.2 > 776.1/758.1/658.1;
- ProB: *m/z* 810.4 > 792.5/676.5/658/5;
- Quinidine: *m/z* 325.2 > 172.0/160/81.2.

Mass-spectrometer was operated under following conditions: Flow rate of drying gas (nitrogen) was 10 L/min, its temperature was 300 °C, flow rate of coating gas (nitrogen) was 11 L/min, its temperature was 350 °C, nebulizer pressure was 50 psi, and the capillary voltage was 3000 V.

This method was validated for qualitative and quantitative analysis of jervine and proA in blood plasma and urine [37]. The linearity was confirmed for concentrations of *Veratrum* alkaloids between 0.1 and 50.0 ng/mL in blood plasma, as well as 0.1 and 100 ng/mL in urine. Also, proB could be detected with this method, while the selectivity for this compound was approved.

## Figures and Tables

**Table 1:** Results of molecular modeling.

Compound	Docking Score (kcal/mol)	Predicted IC_50_ (μmol)	logP	p*K_a_*
Jervine	−10.8	5.00	4.188	9.64
ProA	−6.8	6.18	1.607	7.28
ProB	−6.6	6.20	0.578	7.28
Quinidine	−7.2	7.10 ^1^	3.173	8.71

^1^ Experimental IC_50_ value is 6.90 μmol [21].

**Table 2:** Concentration of *Veratrum* alkaloids in blood plasma of emergency department patients with symptoms of cardiotoxicity.

Patient	*Veratrum* Alkaloid	Alkaloid Concentration, ng/mL				
		0 h	24 h	36 h	48 h	60 h
1	Jervine	0.52	0.14	-	-	-
	ProA	0.20	-	-	-	-
	ProB	+	-	-	-	-
2	Jervine	0.35	0.10	-	-	-
	ProA	0.11	-	-	-	-
	ProB	+	-	-	-	-
3	Jervine	5.01	0.72	0.48	0.33	0.18
	ProA	0.67	-	-	-	-
	ProB	+	+	-	-	-
4	Jervine	0.10	-	-	-	-
	ProA	-	-	-	-	-
	ProB	-	-	-	-	-
5	Jervine	0.15	-	-	-	-
	ProA	0.10	-	-	-	-
	ProB	-	-	-	-	-
6	Jervine	0.11	-	-	-	-
	ProA	-	-	-	-	-
	ProB	-	-	-	-	-
7	Jervine	0.13	-	-	-	-
	ProA	0.12	-	-	-	-
	ProB	+	-	-	-	-

**Table 3:** Concentration of *Veratrum* alkaloids in urine of emergency department patients during detoxification.

Patient	*Veratrum* Alkaloid	Alkaloid Concentration, ng/mL				
		0 h	24 h	36 h	48 h	60 h
1	Jervine	0.58	9.55	-	-	-
	ProA	37.70	1.01	-	-	-
	ProB	+	+	-	-	-
2	Jervine	0.24	1.15	-	-	-
	ProA	6.13	8.14	0.19	-	-
	ProB	+	+	+	-	-
3	Jervine	1.71	5.19	1.85	1.07	0.23
	ProA	4.87	2.81	0.87	0.46	-
	ProB	+	+	+	+	+
4	Jervine	0.23	0.10	-	-	-
	ProA	0.15	-	-	-	-
	ProB	+	+	-	-	-
5	Jervine	0.21	1.47	-	-	-
	ProA	11.96	1.66	-	-	-
	ProB	+	-	-	-	-
6	Jervine	0.46	-	-	-	-
	ProA	54.41	-	-	-	-
	ProB	+	-	-	-	-
7	Jervine	1.39	-	-	-	-
	ProA	52.16	-	-	-	-
	ProB	+	-	-	-	-

**Table 4:** Gradient elution in HPLC-MS/MS analysis.

Time, min	Volume Fraction of Solvent A, %	Volume Fraction of Solvent B, %
0:00	90	10
1:00	90	10
1:10	75	25
1:50	75	25
9:50	50	50
9:60	10	90
11:00	10	90
11:10	90	10
14:00	90	10

## Data Availability

This is not applicable.
